# Erratum: Determination of Low Loss in Isotopically Pure Single Crystal ^28^Si at Low Temperatures and Single Microwave Photon Energy

**DOI:** 10.1038/srep46901

**Published:** 2017-09-20

**Authors:** Nikita Kostylev, Maxim Goryachev, Andrey D. Bulanov, Vladimir A. Gavva, Michael E. Tobar

Scientific Reports 7: Article number: 44813; 10.1038/srep44813 published online: 03 20 2017; updated: 09 20 2017.

The original version of this Article contained typographical errors in the Abstract.

“Whispering Gallery Mode (WGM) analysis revealed large Quality Factors of order 2 × 10^6^ (dielectric loss ~5 × 10^−7^) at high powers, degrading to 7 × 10^−5^ (dielectric loss ~1.4 × 10^−6^ at single photon energy. A very low-loss narrow line width paramagnetic spin flip transition was detected with extreme sensitivity in ^28^Si, with very small concentration below 10^10^ cm^−3^ (less than 10 parts per trillion) and g-factor of 1.995 ± 0.008”.

now reads:

“Whispering Gallery Mode (WGM) analysis revealed large Quality Factors of order 2 × 10^6^ (dielectric loss ~5 × 10^−7^) at high powers, degrading to 7 × 10^5^ (dielectric loss ~1.4 × 10^−6^) at single photon energy. A very low-loss narrow line width paramagnetic spin flip transition was detected with extreme sensitivity in ^28^Si, with very small concentration below 10^11^ cm^−3^ (less than 10 parts per trillion) and g-factor of 1.995 ± 0.008”.

This has now been corrected in the PDF and HTML versions of the Article.

